# Federated learning for COVID-19 mortality prediction in a multicentric sample of 21 hospitals

**DOI:** 10.1371/journal.pcbi.1013695

**Published:** 2025-11-24

**Authors:** Roberta Moreira Wichmann, Murilo Afonso Robiati Bigoto, Alexandre Dias Porto Chiavegatto Filho

**Affiliations:** 1 Brazilian Institute of Education, Development and Research – IDP, Economics Graduate Program, Brasilia, DF, Brazil; 2 University of São Paulo, School of Public Health, São Paulo, SP, Brazil; Northeastern University, UNITED STATES OF AMERICA

## Abstract

We evaluated Federated Learning (FL) strategies for predicting COVID-19 mortality using a multicenter sample of 17,022 patients from 21 diverse Brazilian hospitals. We tested horizontal FL architectures employing Logistic Regression (LR) and a Multi-Layer Perceptron (MLP) via parameter aggregation, alongside a novel Federated Random Forest (RF) using ensemble aggregation. Performance gain (*Δ*AUC, calculated as AUC${\;}_{\text{federated}}$ minus AUC${\;}_{\text{local}}$) was quantified using bootstrap analysis to determine 95% confidence intervals. FL models demonstrated a beneficial collaborative effect. The average *Δ*AUC across the network was +0.0018 for LR, +0.0599 for MLP, and +0.0528 for RF. Crucially, the gain’s magnitude and statistical significance showed a strong inverse correlation with local patient volume (N). Substantial and statistically significant gains concentrated in data-limited institutions (N < 500). For example, the smallest hospital (N=86) achieved a remarkable *Δ*AUC of 0.3682 (95% CI [0.0908, 0.6307]) with the RF model. However, interpreting these benefits requires caution because the 95% CIs for *Δ*AUC crossed zero for the majority of hospitals, suggesting the collaborative model’s statistical advantage is not universally certain at every site. This trade-off was particularly evident with the MLP model which, despite achieving the highest average *Δ*AUC, was the most volatile algorithm, registering the maximum performance degradation in the network (*Δ*AUC = –0.0884, 95% CI [–0.1527, –0.0273]) due to its high sensitivity to local data distribution disparities (non-IID). This study validates FL as an equity-enabling mechanism that effectively enhances predictive capacity where local data scarcity is highest. Our findings underscore that maximizing the most statistically certain benefits of FL requires continuous monitoring and local validation for successful clinical deployment across diverse settings.

## Author summary

The authors, coming from diverse fields of expertise, present a comprehensive analysis of federated learning–a decentralized machine learning approach where multiple institutions collaboratively train a model while keeping data localized to ensure privacy and security. This study evaluates the effectiveness of federated learning architectures that aggregate model parameters through averaging in predicting COVID-19 mortality. By applying this methodology across 21 hospitals throughout Brazil, the study investigates its utility across various patient volume contexts and assesses its predictive performance. Additionally, the authors explore federated models based on decision trees and propose the development of a self-scalable random forest algorithm to enhance predictive capabilities and adaptability. The findings suggest that federated learning holds promise as a powerful solution for predictive challenges in healthcare settings, fostering both innovation and data security.

## Introduction

The development of predictive machine learning algorithms in healthcare presents technical and ethical challenges. First, it must consider the heterogeneous characteristics of the data collected from different centers. Ethically, information privacy should also be closely considered due to the sensitivity of its content. To extract the necessary information and address these issues, a machine learning technique known as federated learning (FL) has been increasingly used. FL is a collaborative decentralized learning model that aims to assign scalability to the training model by using isolated local data from different sources while ensuring information privacy by executing the training model on the data-generating units [1,2]. The technique remotely executes the training model, thereby avoiding information centralization or exchange of patient data between organizations and, consequently, lowers the risk of information leakage or compromise [3].

When running the collaborative training model with data from different sources, the predictive model performance can be improved by interacting with information from various sets of data processed in different institutions [4]. As more data is needed to improve the training and performance of the algorithms, data from isolated sources may be subject to the issue of information dimensionality and under-representation of observations in the data set, creating bias in the predictive results. Thus, the use of a decentralized collaborative learning technique allows the construction of an information network, expanding the dimension of the databases by connecting different repositories while keeping the data in their local sources. The FL method assigns scalability to the training model, generating less biased parameters by using a more complete data distribution [1].

There is a large potential gain of applying FL to predictive analysis of diseases and clinical outcomes, by combining information extracted from patient medical records in different repositories, hospitals, and devices [5]. Pfohl and Huang used the method to predict mortality and length of hospital stay, considering aspects sensitive to the model [6,7]. Lee and colleagues sought similarities between patients to predict patterns of association between clinical outcomes, assisting decision-making [8]. In addition, Brisimi and colleagues used a decentralized learning approach to develop a binary predictive model that classified patients according to their probability of being hospitalized for heart disease [9].

This study aims to use federated learning techniques for health-related predictions using multicentric data from a country with large socioeconomic diversity. We tested different architectures and machine learning algorithm structures, both with federated and standard models, and then compared their predictive performance. This work brings a novel perspective to federated learning by leveraging a large sample of Brazilian hospitals. Given Brazil’s vast ethnic and cultural diversity, federated learning faces an even greater challenge of sample heterogeneity (non-IID), which is addressed here in an unprecedented way.

Furthermore, the study introduces an original contribution by proposing a novel federated learning algorithm based on decision trees, inspired by the scalable Random Forest approach, similar to the work presented by Hauschild and colleagues [10]. Additionally, the article presents a novel hyperparameter optimization strategy for the federated network, offering a unique explanation for the choice of hyperparameter *t*, which determines the number of times the federated network adjusts its parameters and interacts with each hospital.

This study distinguishes itself by tackling the unique challenges of federated learning in a socioeconomically diverse country like Brazil, where hospital resources and patient demographics vary significantly across regions. By analyzing the characteristics of hospitals from all Brazilian regions–including the communities they serve and the resources at their disposal–we underscore the relevance and applicability of our approach. Our findings not only demonstrate the effectiveness of federated learning (FL) in enhancing predictive performance but also provide novel insights into the application of FL in heterogeneous and non-independent and identically distributed (non-IID) settings.

## Materials and methods

### Data base

A cohort of 17,022 patients from 21 different hospitals across all the five regions of Brazil was followed between March and August 2020. The data comes from hospitals associated with the IACOV-BR network coordinated by the Laboratory of Big Data and Predictive Analytics in Health (LABDAPS) of the School of Public Health, University of São Paulo, Brazil. The inclusion criterion for patient data in the study was having a RT-PCR test, regardless of the result. Data collection refers to a 24-hour window before and after the test was performed. The study was conducted in accordance with ethical principles, including compliance with the Brazilian General Data Protection Law (LGPD), and did not involve access to identified patient data.

A total of 22 predictors, as indicated in Table 1, were selected from the variables routinely collected across all hospital, including age, sex, heart rate, respiratory rate, systolic blood pressure, diastolic blood pressure, mean arterial pressure, temperature, hemoglobin, platelets, hematocrit, red blood cell count, mean corpuscular hemoglobin (MCH), red cell distribution width (RDW), mean corpuscular volume (MCV), white blood cells, neutrophils, lymphocytes, basophils, eosinophils, monocytes, and C-reactive protein.

**Table 1:** List of variables used to predict hospitalization mortality in the federated and local learning approaches.

Variables	Description	Unit of Measure
Age	Patient age	Years
Platelets	Platelet count	/mm3
HCM	Mean corpuscular hemoglobin	pg
MCV	Mean corpuscular volume	fL
Leukocytes	Leukocyte count	/mm^3
RDW	Red cell distribution width	%
CRP	C-reactive protein	mg/dL
Basophils	Basophil count	/mm^3
Lymphocytes	Lymphocyte count	/mm^3
Eosinophils	Eosinophil count	/mm^3
Red_Cells_Count	Red blood cell count	4.5 - 6.0 x 10^6/mm^3
Monocytes	Monocyte count	/mm3
Hemoglobin	Hemoglobin level	g/dL
Resp_Rate	Respiratory rate	Breaths per minute
Neutrophil	Neutrophil-to-lymphocyte ratio	Neutrophils/lymphocytes
Hematocrit	Hematocrit level	Percentage of red cells
Heart_Rate	Heart rate	Beats per minute
Sys_Press	Systolic blood pressure	mmHg
Dias_Press	Diastolic blood pressure	mmHg
Mean_Press	Mean arterial pressure	mmHg
Temp	Body temperature	Celsius degrees
Gender	Gender	Categorical

### Statistical analysis

We used the transfer learning architecture Flower to act as a central server to build the global model by aggregating the results of locally trained models from the hospitals [11]. Data preparation packages were created and transmitted to the hospitals, which were then used to define the hyperparameters of the federated learning approach, that served as input for designing strategies for aggregation and parameter connection. Finally, the hyperparameters defined the modeling architecture (models, sampling, and evaluation). The Flower Architecture consists of a cyclical system for updating local data [11]. The server aggregates the parameters (e.g., using Federated Averaging) and returns the updated global model to the hospitals. This process is repeated iteratively until the model converges.

Data from 21 hospitals across all five Brazilian regions were analyzed in this study, with the distribution as follows: North (3 hospitals, n = 1,936), Northeast (4 hospitals, n = 4,325), Midwest (4 hospitals, n = 1,872), Southeast (6 hospitals, n = 7,302), and South (4 hospitals, n = 1,587), as shown in Table 2. Using 22 predictors (Table 1), federated learning approaches were developed to predict mortality among hospitalized patients with COVID-19.

**Table 2:** Hospitals by region in Brazil, with a list of hospitals.

Region	Hospitals by Region
Southeast	Hospital Santa Casa de São Paulo, Hospital das Clínicas da Faculdade de Medicina da USP, Hospital Unimed-Rio, Hospital Universitário Clementino Fraga Filho, Hospital São Francisco de Mogi Guaçu, Hospital Evangélico de Vila Velha - HEVV
Northeast	Hospital Português da Bahia, Hospital Unimed Fortaleza, Hospital Universitário HC de PE, Hospital Universitário Walter Cantídio
North	Fundação Santa Casa de Misericórdia Em Belém, Hospital Santa Julia, Hospital Universitário Getúlio Vargas
Central-West	Hospital Estadual de Luziânia - HEL, Hospital Santa Lúcia, Hospital Universitário Maria Aparecida Pedrossian, Hospital Estadual de Trindade - HETRIN
South	Grupo Hospitalar Conceição, Hospital Moinhos de Vento, Hospital Santa Catarina Blumenau, Hospital Escola da UFPel

We tested the use of a horizontal federated learning system architecture. In this system, the 21 hospitals collaborate to build a final machine learning model through a central server [12]. As presented by Phong and colleagues, we accept the recurring assumption that both the hospitals and the server are honest, disregarding the possibility of leaks from the participant hospitals to the server [13].

**Fig 1:**
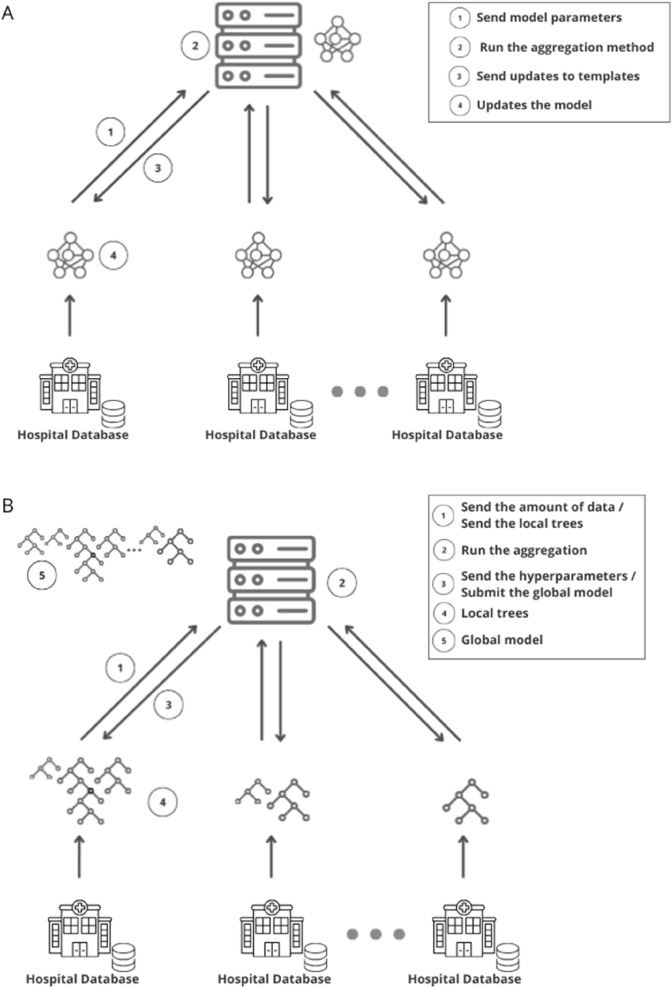
Schematic visualization of the federated learning approaches. (A) Parameter averaging framework (Scenario I): In this system, participants (hospitals) compute model coefficients and send them anonymously to the server for aggregation. The server then returns the updated global model to the participants in an iterative process. (B) Decision tree aggregation framework (Scenario II): Here, the server determines the number of decision trees each hospital should build based on its proportional data size. These locally built trees are then sent back to the server, which aggregates them into a single Federated Random Forest model, subsequently distributed to all participating hospitals.

Generally, the steps for building a federated model can be ordered as follows: locally, participants compute the coefficients of a machine learning model and send them to the server anonymously; the server then aggregates the parameters received from the participants without any learning information from each part; then returns the aggregation results to the participants; and finally the participants update their models with these results [12]. A schematization of this process can be observed in Fig 1A.

We used the AUC-ROC as the metric to measure predictive performance of the federated learning system, according to two scenarios. Scenario I consisted of developing two traditional machine learning models, a Multi Layer Perceptron (MLP) neural network, and Logistic Regression (LR). In this scenario, for both models, the learning process consisted of aggregating the parameters of the models through the Federated Averaging method over the federated system server [14].

The federated learning approach used in Scenario I, as well as its aggregation method, are shown below. The problem formulation requires partitioning the total data among the *K* available clients. Given *n*_*k*_ as the count of data points at client *k* (index set ${𝒫}_{k}$), the overarching objective function, representing the minimized aggregate loss across the federation, is written as:

$$\min_{𝐰\epsilon {\mathbb{R}}^{d}}F(𝐰)=\sum_{k=1}^{K}\frac{n_{k}}{n}F_{k}(𝐰)\;\text{where}\;F_{k}(𝐰)=\frac{1}{n_{k}}\sum_{i\in {𝒫}_{k}}f_{i}(𝐰)$$ (1)

Here, *n* is the total number of samples across all hospitals; **w** represents the model parameters and *f*_*i*_ is the loss function for the *i*-th data sample [14].

The Federated Averaging algorithm, shown in Algorithm 1, is used in Scenario I and implements the aggregation-based learning logic described in Equation 1. It can be summarized and schematized as follows: The *K* hospitals are indexed by *k*; *B* represents the local minibatch size, *E* denotes the number of local epochs, and *η* is the learning rate [14].

**Algorithm 1 Federated averaging algorithm.** The number *K* of hospitals is indexed by *k*; *B* is the size of the local data samples, *E* represents the number of local training steps, *η* is the learning rate, and *C* represents a fraction of the *K* sample, which can be considered as *C* = 1 in the context of this work [14].

1: **Server executes:**

2: Initialize ${𝐰}_{0}$

3: **for** each round t=1,2,… **do**

4:   m←max(C·K,1)

5:   $S_{t}\leftarrow$ (random set of *m* hospitals)

6:   **for** each hospitals $k\in S_{t}$ in parallel **do**

7:    ${𝐰}_{t+1}^{k}\leftarrow \text{HospitalUpdate}(k,{𝐰}_{t})$

8:   **end for**

9:   $m_{t}\leftarrow \sum_{k\in S_{t}}n_{k}$

10:   ${𝐰}_{t+1}\leftarrow \sum_{k\in S_{t}}\frac{n_{k}}{m_{t}}{𝐰}_{t+1}^{k}$

11: **end for**

——————————————————————–

**HospitalUpdate** called during the update of *w*_*t* + 1_ in the process shown above

——————————————————————–

12: **HospitalUpdate**(k, **w**):

13: **// Run on hospital *k***

14: ℬ← (split *P*_*k*_ into batches of size *B*)

15: **for** each local epoch *i* from 1 to *E* **do**

16:   **for** batch b∈ℬ **do**

17:    𝐰←𝐰−η∇ℒ(𝐰,b)

18:   **end for**

19: **end for**

20: **return w** to server

To determine the number of training epochs for both MLP and LR, we conducted an analysis using a validation dataset. This analysis focused on monitoring the convergence of the AUC metric, which guided the selection and justification of the hyperparameter *t* in Algorithm 1.

Our approach to empirically observing and adopting this hyperparameter is original and has not been previously documented in the literature. Existing studies suggest that setting this hyperparameter to *t* = 10 is generally sufficient for the convergence of federated networks [15,16]. However, other authors, such as Soltan *et al*., have incorporated a significantly higher number of training epochs, using *t* = 150 [17]. As in our previous setup, we adopted the Flower Architecture, which integrates both a secure parameter transmission strategy from participants to the server and the Federated Averaging Aggregation method [11].

Since the objective of this work was to evaluate the predictive capacity of the models from a federated learning perspective, the hyperparameters of the models were not extensively explored. For the Multilayer Perceptron (MLP), TensorFlow and Keras were used, and the architecture was designed with two hidden layers to balance model complexity and computational efficiency, following best practices for neural network design. The first hidden layer consists of 20 units, and the second hidden layer consists of 10 units, which allows the model to capture hierarchical patterns in the feature space without overfitting, especially given the moderate dimensionality of the input data. To measure the MLP loss function, categorical cross-entropy was used and the Adam method was selected as the optimizer. For LR, Scikit-learn was used with its standard hyperparameters, and an l2 penalty [18].

In Scenario II, a federated model based on Random Forests was developed and evaluated. The central idea behind this scenario is to aggregate decision trees built in each hospital during the federated learning process. Unlike Scenario I, which explicitly uses the mathematical aggregation presented in Equation 1 and Algorithm 1, Scenario II draws inspiration from the principles of Equation 1 to proportionally incorporate decision trees based on the amount of data available at each hospital. Instead of weighting entire random forests from each hospital, as proposed by Hauschild and colleagues, this work introduces a novel approach where the number of trees to be built at each hospital is predefined and proportional to the size of its local dataset [10]. This strategy not only reduces computational overhead in hospitals with fewer patients but also ensures scalability in processing and computational costs.

The process begins with the initialization of the server, which identifies the number of participating hospitals, denoted as *K*. Each hospital is anonymously indexed by a unique identifier *k*. The server then requests and receives, in an anonymized manner, the number of patients from each hospital. Based on this information, the server calculates the number of decision trees each hospital must develop, proportional to its local dataset size. The total number of trees *M* is a hyperparameter defined during server initialization and remains fixed throughout the federated learning process. In this study, *M* was set to 550 to ensure consistency and computational efficiency.

Once the server determines the number of trees for each hospital, it sends this information back to the respective *k*. Each hospital then processes its local data using a preprocessing script (prep_iacov.py) and develops the required number of decision trees. To enhance privacy and security, the training process incorporates bootstrapping and bagging techniques, which randomly sample both features and patients. This approach not only improves the robustness of the model but also complicates potential reverse engineering attacks aimed at reconstructing the training data.

After developing the trees, each hospital sends them back to the server, still anonymized and indexed by *k*. The server aggregates these trees into a single Federated Random Forest by shuffling and combining them. This global model is then distributed back to all participating hospitals for evaluation. The performance of the federated model is assessed in two stages: (1) each hospital evaluates its local model before sending it to the server, and (2) each hospital evaluates the global model using its local test data. This two-stage evaluation allows for a comprehensive comparison between local and federated learning outcomes.

The methodology developed for Scenario II minimizes communication overhead by reducing the number of information exchanges between the server and participating hospitals. This addresses a common challenge in federated learning, as highlighted by Hauschild *et al* [10]. Additionally, the approach enhances security by transmitting entire models (trees) rather than raw parameters, reducing the risk of data exposure [15]. Scenario II employs Algorithm 1 as its foundation, adapting it for the construction of decision trees instead of updating parameters of traditional models. Initially, the server selects all hospitals (*C* = 1) to participate in the training process, where each hospital *k* executes the *HospitalUpdate* procedure, building decision trees proportionally to the size of its local dataset *P*_*k*_.

For this process, a Python library was developed by the authors, which includes specific modules for the server and participants. The library simplifies the implementation of federated learning, requiring only two lines of code to initialize the server and instantiate the participating hospitals. This user-friendly design enables research groups to simulate and employ the methodology with minimal effort. Fig 1B presents a schematic of the federated structure.

For the evaluation of the results from the three federated machine learning models, the AUC-ROC metric was used to measure the predictive capacity of the models and to assess the performance gain compared to the local learning context within the same hyperparameter space. The data was divided into training and test sets, with 80% of the data used for training and 20% reserved for testing. This split was chosen to ensure a robust evaluation of model performance while maintaining sufficient data for training. Importantly, the AUC-ROC results for both local and federated learning were calculated using the same test set, ensuring a fair comparison of model performance. The test set was exclusively used during the evaluation phase and was not involved in the training process, thereby providing an unbiased assessment of the models’ predictive capabilities.

To empirically validate and compare the performance of the local and federated models, we implemented a standardized computational pipeline in Python. For each of the 21 participating hospitals, a local model was trained exclusively on its own dataset to serve as a performance baseline. Subsequently, the corresponding pre-trained federated model, developed under either Scenario I or II, was loaded for direct comparison. We employed a bootstrap analysis with 1000 iterations to ensure statistical robustness and to estimate the uncertainty of the AUC-ROC.

In each iteration, a new test set was generated by sampling with replacement from the original test set. The AUC-ROC was then calculated for both the local and federated models on this resampled data, and the difference between them ($\Delta AUC=AUC_{\text{federated}}-AUC_{\text{local}}$) was recorded. This procedure yielded empirical distributions for the local AUC, federated AUC, and *Δ*AUC for each hospital. From these distributions, we calculated the mean performance and 95% confidence intervals (CI).

The performance gain of the federated model was quantified by the mean *Δ*AUC and its 95% confidence interval, where an interval composed entirely of positive values indicates a consistent advantage over the local model. The final results were consolidated into a summary table and visualized to explore the relationship between performance gain and the number of patients per hospital.

All codes developed for the study, which were built for this methodology, are available in https://github.com/labdaps/federated-learning-for-health-in-a-multicentric-sample-of-hospitals.git. The results of the AUC-ROC metric for each hospital subjected to the learning process can also be found in the code repository.

## Results

While the explicit tuning of communication rounds (*t*) is not extensively explored in the federated learning literature, we performed a convergence analysis to rigorously determine this hyperparameter. This analysis, presented in Supporting Information (S1 Fig), plots the AUC-ROC performance on a server-side validation set against each communication round. This methodological approach enabled us to identify an optimal t that maximizes predictive performance without incurring unnecessary computational costs, addressing a critical aspect of model efficiency.

In Scenario II, *t* is inherently set to 1 by the engineering structure of the developed algorithm. For Scenario I, a rapid convergence of the AUC-ROC metric is observed for both Logistic Regression (LR) and Multilayer Perceptron (MLP) algorithms. The graph analysis reveals that, around 5 iterations, the algorithms achieve significant stability in the performance of the global federated model. This behavior justifies the adoption of *t* = 5 in the proposed methodology, as higher values do not yield substantial improvements in the metric but may increase computational costs.

Furthermore, it is important to emphasize that the hyperparameter *t* is intrinsically linked to the processing cost of the federated network. Its careful exploration, as conducted in this study, is crucial to avoid unnecessary computational expenses, processing overload in participating hospitals, and potential losses of connection to the federated network. The approach proposed here demonstrates that selecting *t* = 5 provides an optimal balance between predictive performance and computational efficiency, contributing to the practical feasibility of implementing FL in multicentric environments.

**Fig 2:**
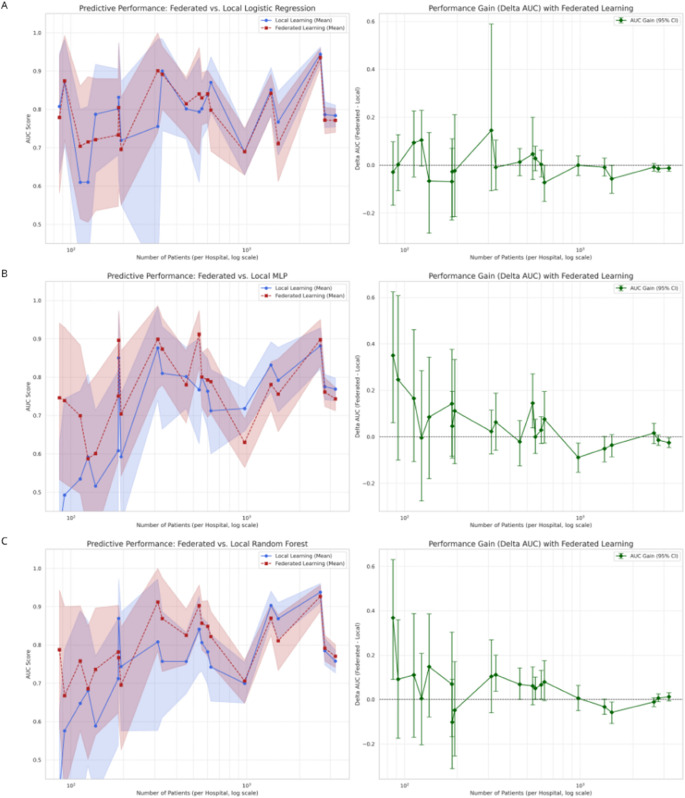
Comparison of predictive performance between federated and local learning models. The figure illustrates the performance results for three machine learning models: (A) Logistic Regression, (B) Multi-Layer Perceptron (MLP), and (C) Random Forest. For each model, the left panel compares the mean Area Under the Curve (AUC) of the federated model (red dashed line) against the locally trained model (blue solid line), with shaded areas representing the 95% confidence intervals derived from bootstrap analysis. The right panel displays the performance gain, quantified as the Delta AUC (*Δ*AUC = AUC${\;}_{\text{federated}}$ - AUC${\;}_{\text{local}}$), where the error bars indicate the 95% CI. The horizontal dotted line represents a performance gain of zero. The x-axis for all plots is on a logarithmic scale and corresponds to the number of patients at each hospital. A consistent trend is observed across all models where federated learning generally outperforms local learning, an advantage that is particularly evident for hospitals with smaller datasets.

Fig 2 presents a comparative analysis of predictive performance, measured by the AUC-ROC metric, between the federated and local learning paradigms for the three investigated algorithmic models. The evaluation, stratified by each of the 21 participating hospitals, elucidates a complex relationship between model performance and local dataset size (N), presented on a logarithmic scale.

The results reveal a trend where federated models, in general, tend to exhibit superior predictive performance compared to their locally trained counterparts. However, the magnitude and statistical significance of this advantage show an inverse correlation with the dataset size of each hospital. For institutions with limited patient cohorts (e.g., N < 500), collaboration in the federated network frequently results in a substantial performance increment. For example, as detailed in S2 and S3 Tables, the hospital with the smallest cohort (N=86) achieved statistically significant performance gains for the MLP (*Δ*AUC=0.3507, 95% CI [0.0606, 0.6243]) and Random Forest (*Δ*AUC=0.3682, 95% CI [0.0908, 0.6307]) models. Conversely, for hospitals with vast data repositories (e.g., N > 1,000), the performance differential attenuates considerably. S1 and S2 Tables indicate that for the two largest centers, the local Logistic Regression and MLP models showed a marginal, yet statistically significant, advantage over the federated models, suggesting a point of local data sufficiency at which the benefit of federated aggregation becomes less pronounced.

The Logistic Regression model (Fig 2A and S1 Table), serving as a linear baseline, exhibited marginal and inconsistent performance gains. While 9 of the 21 hospitals recorded a positive mean *Δ*AUC, the wide confidence intervals, which frequently intersected the null threshold, suggest that simple parameter averaging of a linear model offers a limited, often statistically insignificant, benefit. This approach establishes a performance floor but appears insufficient to fully harness the complex patterns available in the distributed data.

In sharp contrast, the MLP (Fig 2B and S2 Table), a non-linear model, demonstrated a pronounced performance dichotomy, underscoring both the potential and the perils of federated learning. For institutions where local data was insufficient to train a complex model effectively, the federated approach provided a pathway to high performance by leveraging the collective dataset. However, this same complexity rendered the model susceptible to negative knowledge transfer, yielding the most significant performance degradation in certain hospitals where the global model’s parameters failed to generalize to a unique local data distribution (e.g., Hospital Estadual de Luziânia, *Δ*AUC=–0.0884, 95% CI [–0.1527, –0.0273]). This volatility highlights the model’s acute sensitivity to non-IID data.

Finally, the Random Forest model (Fig 2C and S3 Table), implemented via the distinct tree-aggregation architecture of Scenario II, offered the most consistently robust performance. By constructing a global ensemble from locally trained decision trees rather than averaging abstract parameters, this method inherently mitigates the impact of client data heterogeneity, as a single, divergent local dataset is less likely to corrupt the entire model. This structural resilience is evidenced by 16 of the 21 hospitals showing a positive mean *Δ*AUC. Notably, the performance improvements were statistically significant for a subset of these institutions, which included hospitals with varying cohort sizes. This suggests that for heterogeneous healthcare data, ensemble-based aggregation may provide a more stable and reliable pathway to performance enhancement in federated networks.

Across the network, a substantial range of performance gains (*Δ*AUC) was observed, depending on the algorithm and local data size (S1-S3 Tables). Overall, the average *Δ*AUC across all 21 hospitals was +0.0018 for Logistic Regression (LR), +0.0599 for the Multi-Layer Perceptron (MLP), and +0.0528 for the Random Forest (RF).

In summary, the empirical findings suggest a diminishing marginal utility of federated learning as local data sufficiency increases. The primary beneficiaries of the collaborative architecture are institutions with smaller datasets, for which federated learning allows them to transcend local statistical limitations and access a more generalizable and robust model. The results, therefore, not only validate the potential of federated learning as a mechanism for enhancing predictive capacity in heterogeneous healthcare networks but also underscore that its application does not guarantee universal superiority, with its impact being contingent upon the intrinsic characteristics of each participating node.

To systematically investigate the relationship between the socioeconomic context and the performance of federated learning, an aggregated analysis at the regional level was performed, with the results consolidated in S4 Table. The comparison of the rank order of regional per capita income with that of the average hospital sample size in our study reveals a partial correlation. While the Southeast region shows high values in both indicators, the South (high income, low average sample size in the study) and Northeast (low income, high average sample size in the study) regions demonstrate that intra-regional heterogeneities and the specific profile of the participating hospitals influence this relationship.

The main finding of this analysis is a strong inverse correlation between the average hospital sample size per region and the mean performance gain (*Δ*AUC) obtained with the non-linear federated learning models (MLP and Random Forest). The Central-West and South regions, which featured the smallest average number of patients per hospital in our sample (468 and 397, respectively), correspondingly exhibited the highest average performance gains with the robust Random Forest model (+0.1309 and +0.0950). In contrast, the Southeast region, with the largest average cohort (1,217), registered the most modest gain (+0.0245). Notably, the Logistic Regression model did not show the same pattern, with marginal performance gains across the regions, indicating that model complexity is a key factor for leveraging the benefits of federated aggregation.

It is important to contextualize that these regional averages are derived from the study’s specific sample and, therefore, may not fully represent the overall distribution of hospital sizes in each region. Nevertheless, the consistency of the observed pattern suggests that the performance gain with federated learning is more strongly associated with an institution’s local data scarcity than with regional socioeconomic factors in isolation. This finding highlights the potential of the approach to benefit institutions with smaller data cohorts, regardless of their geographical location.

## Discussion

This study provides an empirical evaluation of federated learning (FL) for clinical prediction within a large-scale, multicentric healthcare network, uniquely characterized by the profound socioeconomic and data heterogeneity that mirrors global health challenges. Our findings demonstrate that while FL offers a promising paradigm to overcome the limitations of data silos, its efficacy is critically modulated by institutional data volume, algorithmic architecture, and the inherent statistical uncertainty of collaborative modeling. This discussion offers a critical interpretation of these findings, their practical and theoretical implications, and their contribution to the advancement of equitable artificial intelligence in real-world clinical settings.

Although the average performance gains observed with federated learning (FL) are notable, a careful interpretation of the 95% CIs reveals an important limitation. As illustrated in Fig 2, the CIs for most hospitals frequently intersect the null threshold (*Δ*AUC = 0), indicating that improvements at the individual institution level were often not statistically significant. This observation does not invalidate the approach but rather contextualizes it: federated learning provides a global model that, on average, tends to outperform local models, yet the certainty of this advantage varies across institutions. From a clinical and operational standpoint, this suggests that the federated model should be regarded as an enhanced baseline–one that generally offers better overall performance but may still require local validation before being adopted as a definitive predictive solution.

Across the three algorithms evaluated, this nuanced pattern was consistently observed. Federated models achieved higher mean AUCs compared to locally trained models, particularly in smaller hospitals (N < 500). For instance, institutions with limited data demonstrated statistically significant performance gains, such as the smallest hospital (N = 86), which achieved *Δ*AUC = 0.3507 (95% CI [0.0606, 0.6243]) with the MLP and *Δ*AUC = 0.3682 (95% CI [0.0908, 0.6307]) with the Random Forest. Conversely, in larger centers, these differences diminished and, in some cases, even favored local models, reflecting a saturation effect related to data sufficiency. Thus, while FL effectively mitigates data scarcity, its marginal benefit decreases as local datasets become sufficiently large and diverse to train competitive models independently.

For the Logistic Regression model (Fig 2A), performance gains were modest and statistically inconsistent, as most 95% CIs crossed the null line. This behavior aligns with expectations for linear models, where simple parameter averaging across heterogeneous sites provides limited representational benefit. In contrast, the MLP model (Fig 2B) exhibited greater variability, in which some hospitals achieved substantial and statistically significant improvements, whereas others experienced minor yet significant performance declines. These findings illustrate both the potential and the fragility of federated deep learning–highly effective when data heterogeneity is moderate, but susceptible to negative transfer when data distributions differ sharply across sites.

The Random Forest model (Fig 2C) demonstrated the most stable and statistically reliable results, with 16 out of 21 hospitals showing positive mean *Δ*AUCs and several with 95% CIs entirely above zero. The tree-based aggregation mechanism likely contributes to this robustness, as the ensemble structure mitigates the propagation of bias from individual nodes. These characteristics make Random Forest-based federated strategies particularly appealing in healthcare applications, where institutional data heterogeneity is the norm rather than the exception.

Overall, these results call for cautious optimism regarding the use of federated learning in multicentric healthcare environments. While the aggregate evidence supports its ability to improve predictive performance without centralizing sensitive data, the wide and frequently overlapping confidence intervals emphasize that such improvements are probabilistic rather than absolute. In practical terms, federated models can serve as strong, privacy-preserving baselines that enhance overall performance across institutions; however, their superiority at the individual hospital level is not universally guaranteed. Continuous monitoring, local calibration, and adaptive aggregation strategies remain essential to ensure that the global benefits of FL effectively translate into consistent clinical improvements across diverse healthcare settings.

The regional analysis (Table S4) revealed that performance gains from federated learning (FL) varied substantially across Brazil’s macro-regions. A clear inverse relationship was observed between the average hospital sample size and the mean performance gain of the non-linear models. The Central-West and South regions, with the smallest average hospital cohorts (468 and 397 patients, respectively), achieved the largest gains with the Random Forest model (+0.1309 and +0.0950), while the Southeast–home to the largest cohorts (1,217 patients per hospital)–showed only modest improvements (+0.0245). This pattern indicates that FL particularly benefits institutions with limited local data, enhancing model generalization when local training data are scarce.

Regarding the socioeconomic context, no consistent relationship emerged between regional wealth or healthcare infrastructure and FL performance. For instance, the Southeast and South–regions with the highest per capita income (R$ 2,127 and R$ 2,018), HDI (0.803 and 0.811), and physician density (3.73 and 3.23 per 1,000 inhabitants)–did not consistently outperform less affluent regions. Conversely, regions with fewer resources but smaller hospital datasets (e.g., Central-West) achieved the largest performance gains. These findings suggest that data quantity and representativeness, rather than socioeconomic context alone, are the main drivers of FL benefits.

A key limitation of this analysis lies in the representativeness of the study sample. The number and size of hospitals per region do not reflect Brazil’s national hospital distribution, and local differences in data quality and case mix likely influenced the observed outcomes. Additionally, the regional socioeconomic indicators used here (e.g., income, HDI, physician density) are aggregated measures that may obscure within-region inequalities. Therefore, while the regional patterns are informative, they should be interpreted as exploratory rather than conclusive evidence of socioeconomic influence on FL performance.

This study differentiates itself from similar work, such as the analysis by Pfohl and colleagues, through its unique focus on real-world heterogeneity and socioeconomic context within a large-scale Brazilian public health network. While both studies applied FL to mortality prediction, our work across 21 Brazilian hospitals using varied architectures and highly heterogeneous data provides distinct insights. Pfohl *et al*. found that FL models performed comparably to centralized learning but encountered challenges integrating differential privacy, resulting in performance reduction. Conversely, our findings focus on the critical role of data volume and demonstrate FL’s ability to significantly outperform local training, especially in data-limited settings. This contrast highlights our study’s unique contribution: demonstrating the practical benefits and algorithmic trade-offs of FL as a lever for health equity in resource-constrained environments, while underscoring the universal challenge of balancing robust model performance with strict privacy guarantees.

## Limitations of the study

Although we included hospitals from all regions of Brazil, our results were limited by the uneven distribution of patients across the different regions. This limitation may have impacted the representativeness of some regions of the country, and therefore our results should be interpreted with caution, considering the non-IID nature of the data. Another important limitation is that the selected hospitals were disconnected and independent, which may have led to differences in local medical procedures and data collection protocols, potentially introducing biases in the data. While federated learning helps mitigate these biases by aggregating models without centralizing data, variations in data quality and missing data across hospitals remain a challenge. Additionally, the computational and communication overhead of federated learning, particularly in hospitals with limited resources, posed a significant constraint. To address this, we optimized hyperparameters and reduced the number of iterations to balance performance and efficiency.

Regarding data privacy and security, while the study does not explicitly use encryption, the federated learning approach inherently minimizes the need for this by sharing only model parameters rather than raw data. However, secure communication protocols are likely needed in real-world settings to protect the transmission of these parameters. The decentralized nature of federated learning aligns with ethical considerations, as it preserves patient privacy and complies with the Brazilian General Data Protection Law (LGPD). Nonetheless, further exploration of the ethical implications, including the potential for re-identification attacks and the robustness of privacy-preserving mechanisms, is warranted. These limitations highlight the need for continued research to refine federated learning approaches in healthcare, ensuring both predictive accuracy and ethical compliance.

## Conclusion

This study provided a robust, multicentric empirical evaluation of Federated Learning (FL) applied to hospital mortality prediction within a context defined by profound data and socioeconomic heterogeneity, characteristic of the Brazilian public health network. Our methodology successfully leveraged a horizontal FL architecture across 21 hospitals and 17,022 patients, comparing the performance of three distinct algorithms–Logistic Regression, MLP, and Random Forest–under parameter and model-based aggregation strategies. The results consistently demonstrated that the marginal benefit of FL is inversely correlated with local data sufficiency. While the mean performance gain (*Δ*AUC) was positive across the network, the statistically significant advantages were predominantly concentrated in data-limited hospitals (N < 500), establishing FL as an effective mechanism for mitigating data scarcity. This finding underscores FL’s potential as a tool for health equity, allowing institutions with limited local cohorts to access more generalizable and robust predictive models. The comparative analysis of algorithmic stability highlighted the Random Forest model (utilizing a tree-based aggregation strategy) as the most reliable architecture for this non-IID setting, demonstrating superior resilience to heterogeneity compared to the volatile MLP and the statistically inconsistent Logistic Regression. Furthermore, our regional analysis found that institutional data quantity, rather than regional socioeconomic wealth or infrastructure, was the dominant driver of performance gains. This work’s primary contribution lies in validating the algorithmic trade-offs and practical viability of FL within a highly heterogeneous real-world health network, offering a critical foundation for the ethical deployment of privacy-preserving AI. Future research must build upon this foundation by exploring personalized FL strategies to ensure that the aggregate network benefits translate into guaranteed, consistent performance improvements at every participating institution.

## Supporting information

S1 Fig Evolution of the hyperparameter *t* in relation to the LR and MLP models. This figure illustrates the convergence of the AUC-ROC metric across iterations of the hyperparameter *t* for the Logistic Regression (LR) and Multilayer Perceptron (MLP) models in the federated learning framework. The graph demonstrates how the predictive performance stabilizes as *t* increases, with significant convergence observed around 5 iterations. This analysis highlights the importance of hyperparameter tuning to balance model performance and computational efficiency in federated learning. This figure was developed by the authors.
(TIFF)

S1 Table Raw performance metrics for the Logistic Regression (LR) model. The table presents the raw output from the bootstrap analysis, showing the mean, lower, and upper confidence interval bounds for the AUC of local and federated models, and the performance gain (*Δ*AUC). For each of the 21 participating hospitals, the table lists the mean Area Under the Curve (AUC) and its 95% confidence interval (CI) for both the locally trained and the federated models. The final columns quantify the performance gain via the mean Delta AUC (*Δ*AUC) and its 95% CI, providing a direct, hospital-by-hospital comparison between the two learning approaches for this linear model.
(XLSX)

S2 Table Raw performance metrics for the MLP model. The table presents the raw output from the bootstrap analysis, showing the mean, lower, and upper confidence interval bounds for the AUC of local and federated models, and the performance gain (*Δ*AUC). It provides a hospital-level breakdown of the mean AUC and 95% CI for the local and federated MLP models. The table also includes the mean *Δ*AUC and its confidence interval, allowing for an assessment of the performance gain achieved by the federated neural network.
(XLSX)

S3 Table Raw performance metrics for the Random Forest (RF) model. The table presents the raw output from the bootstrap analysis, showing the mean, lower, and upper confidence interval bounds for the AUC of local and federated models, and the performance gain (*Δ*AUC). Similar to the preceding tables, it presents the mean AUC and 95% CIs for the local and federated models at each hospital. The performance gain, measured by the *Δ*AUC and its 95% CI, is also shown, offering a detailed comparison for this ensemble-based learning method.
(XLSX)

S4 TableRegional analysis of socioeconomic indicators and federated learning performance gain.The table presents a systematic comparison across Brazil’s five macro-regions, linking key socioeconomic and health indicators with the study’s sample characteristics (number of hospitals and average patient cohort size per hospital). The primary outcome, the mean performance gain from federated learning (*Δ*AUC = AUC${\;}_{\text{federated}}$ - AUC${\;}_{\text{local}}$), is presented for each of the three models: Logistic Regression (LR), Multilayer Perceptron (MLP), and Random Forest (RF). The mean *Δ*AUC values for each region were calculated by averaging the hospital-specific mean *Δ*AUCs reported in S1 Table (for Logistic Regression), S2 Table (for MLP), and S3 Table (for Random Forest) across all hospitals within that geographical region. This allows for a higher-level investigation of the relationship between regional characteristics and the benefits of the federated approach.(XLSX)
