# Supplementary figures and images for: Federated learning for COVID-19 mortality prediction in a multicentric sample of 21 hospitals
Source: PLoS Comput Biol. 2025 Nov 24;21(11):e1013695. doi: 10.1371/journal.pcbi.1013695 (PMC12643281; doi:10.1371/journal.pcbi.1013695)

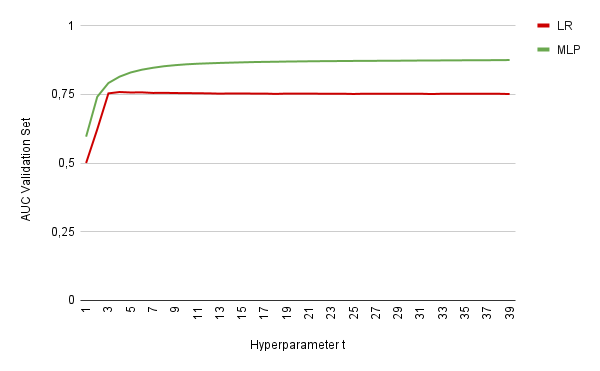

Supplement: S1 Fig — This figure illustrates the convergence of the AUC-ROC metric across iterations of the hyperparameter t for the Logistic Regression (LR) and Multilayer Perceptron (MLP) models in the federated learning framework. The graph demonstrates how the predictive performance stabilizes as t increases, with significant convergence observed around 5 iterations. This analysis highlights the importance of hyperparameter tuning to balance model performance and computational efficiency in federated learning. This figure was developed by the authors. (TIFF) [file pcbi.1013695.s001.tif]
